# Message Delivery for the Treatment of Posttraumatic Stress Disorder: Longitudinal Observational Study of Symptom Trajectories

**DOI:** 10.2196/15587

**Published:** 2020-04-29

**Authors:** Matteo Malgaroli, Thomas Derrick Hull, Shannon Wiltsey Stirman, Patricia Resick

**Affiliations:** 1 NYU Grossman School of Medicine New York, NY United States; 2 Columbia University New York, NY United States; 3 Talkspace New York, NY United States; 4 Standford University Stanford, CA United States; 5 National Center for PTSD Washington DC, DC United States; 6 Duke University Durham, NC United States

**Keywords:** PTSD, telemedicine, messaging, textmessaging, psychotherapy, telehealth, digital health

## Abstract

**Background:**

Individuals with posttraumatic stress disorder (PTSD) face symptoms that can hinder access to treatment, such as avoidance and guilt. Telemedicine offers a technological solution to increase access to mental health care and overcome barriers to treatment. Although an increasing body of literature focused on synchronous telehealth (eg, live video), no studies have examined the delivery of PTSD treatment via two-way multimedia messages (ie, texting or messaging).

**Objective:**

The aim of this study was to conduct a longitudinal observation of treatment for PTSD delivered using two-way asynchronous messaging. We also sought to identify individual and treatment characteristics that could predict the observed outcome differences.

**Methods:**

Outpatients diagnosed with PTSD (N=475) received interventions from licensed therapists, which were delivered via messaging once or more than once per day, 5 days a week for 12 weeks. PTSD symptoms were assessed every 3 weeks using the PTSD Checklist for Diagnostic and Statistical Manual of Mental Disorders-5. Trajectories of PTSD symptoms were identified using growth mixture modeling (GMM). Using logistic regression, the demographic, treatment, and messaging characteristics of patient groups that improved were compared with the characteristics of patient groups that did not improve.

**Results:**

The GMM identified 4 trajectories of PTSD symptoms: *moderate improvement* (197/475, 41.4%), *high symptoms* (197/475, 41.4%), *chronic symptoms* (61/475, 12.9%), and *acute improvement* (20/475, 4.3%). Patients with a clinically significant reduction in PTSD symptoms (231/475, 48.6%) were more likely to communicate via video (odds ratio [OR] 1.01, 95% CI 1.01-1.05; *P*=.03), have a higher working alliance with their therapist (OR 1.03, 95% CI 1.01-1.05; *P*=.02), and be at their first treatment experience (OR 2.03, 95% CI 1.18-3.54; *P*=.01). Treatment adherence was associated with greater therapeutic alliance (OR 1.07, 95% CI 1.03-1.10; *P*<.001), education (OR 2.13, 95% CI 1.13-4.03; *P*=.02), and more patient-generated messages per week (OR 1.08, 95% CI 1.04-1.13; *P*<.001).

**Conclusions:**

Multimedia message delivery for PTSD treatment showed symptom-reduction rates similar to traditional forms of treatment delivery, suggesting further study of messaging as a treatment medium. Most patients completed an 8-week course, reflecting the acceptability of messaging interventions. Delivering treatment via two-way messaging offers increased opportunities for widespread access to mental health care.

## Introduction

Over the course of a lifetime, most people are exposed to at least one potentially traumatic event [1], and approximately 9.7% of women and 3.6% of men [2] will develop posttraumatic stress disorder (PTSD) [3]. PTSD symptoms can be extremely debilitating and are associated with significant functional impairment in education, childbearing, relationships, and financial income [4]. Moreover, lifetime prevalence of PTSD comorbidity has been estimated at approximately 80% [5,6], with higher rates for substance abuse, depression, and anxiety [7]. Comorbidity, in turn, greatly increases day-to-day functional impairment and disability [8-10].

Although many evidence-based treatments for PTSD have been found to be efficacious [11,12], access to face-to-face treatment is not so straightforward. In addition to traditional barriers to treatment, including cost, insurance, stigma, and physical impairments [13-15], individuals with PTSD also face additional hindrances such as avoidance and shame that often result in isolation from their communities. Geographic remoteness can also prevent access to care, and in areas with ongoing violence (and consequently high incidence of PTSD), it can be dangerous for mental health professionals to practice [16]. These obstacles tend to reduce seeking and obtaining of treatment, perpetuating the economic and social costs of untreated illness [17]. Given the particularly debilitating impact of PTSD symptoms, it is imperative to improve rates of treatment access.

Technological delivery is a solution to removing socioeconomic barriers to treatment. Telemedicine enables synchronous (eg, videoconferencing) and asynchronous (eg, texts, images, audio recordings, and video recordings) interactions and is rapidly growing in popularity. Multiple studies have shown the equivalence or noninferiority of PTSD treatment via videoconferencing compared with in-person treatment [18-21]. Other types of technology have also shown encouraging results, such as virtual reality [22], web-based text-based interventions [16,23-25], and email [26].

Although mostly used as an adjunct to treatment [27], asynchronous two-way modalities such as multimedia message service (MMS) have emerged as a potentially primary means of treatment delivery. Owing to its wide population reach, MMS can provide accessible mental health treatment to large numbers of individuals [28], including those in challenging geographic contexts. Early studies suggested that treatment via two-way messaging (ie, texting) delivers outcome rates comparable with other methods for individuals with a wide range of diagnoses [29]. However, the effect of therapy via messaging has not been investigated in a large PTSD population.

In this study, we conducted a longitudinal observation of the delivery of PTSD treatment via two-way multimedia messaging (ie, text, audio, and video). Consistent with once-weekly face-to-face therapy standards, we examined a period of 12 weeks of treatment. Importantly, the study took place in an ecologically valid outpatient telemedicine setting, portraying how two-way messaging would be used in naturalistic contexts. To our knowledge, this is the first study to examine the course of messaging telemedicine treatment for PTSD. Our first aim was to examine overall rates of PTSD reduction and then identify heterogeneous trajectories of symptom changes during treatment. We also sought to identify individual and treatment characteristics that could predict the observed outcome differences.

## Methods

### Participants and Setting

Our sample consisted of 18- to 65-year-old treatment-seeking individuals in the United States, who signed up for a web-based therapy platform (Talkspace). The platform is accessible through internet search, through employee assistance programs, and, as a behavioral health benefit, through some individual insurances. Patients first underwent a biopsychosocial intake interview with a licensed therapist through a live messaging system. Presenting complaints, diagnosis, treatment goals, treatment history, and provider preferences were determined by the clinician during the assessment. Patients then chose a therapist for their treatment from a selected assortment of clinicians, licensed in the state where the patients resided. The matching algorithm selection was based on intake information, therapists’ previous treatment outcomes, and patients’ preferences (eg, the therapist’s gender). The study used archival data under the platform Terms of Use and was approved by the institutional review board at Columbia University Teachers College.

The inclusion criteria were as follows: PTSD diagnosis confirmed by clinical intake and working diagnosis with a licensed therapist, initial PTSD symptom score of 33 or above as measured by the PTSD Checklist for Diagnostic and Statistical Manual of Mental Disorders-5 (PCL-5), and regular internet and/or phone access. The exclusion criteria were as follows: comorbid bipolar or psychotic-spectrum disorders (established by clinical intake/interview); comorbid substance or alcohol abuse (established by clinical intake/interview); and suicidal ideation, intent, plan, and/or behavior requiring higher level of care, as measured by the Columbia Suicide Severity Rating Scale.

**Figure 1:**
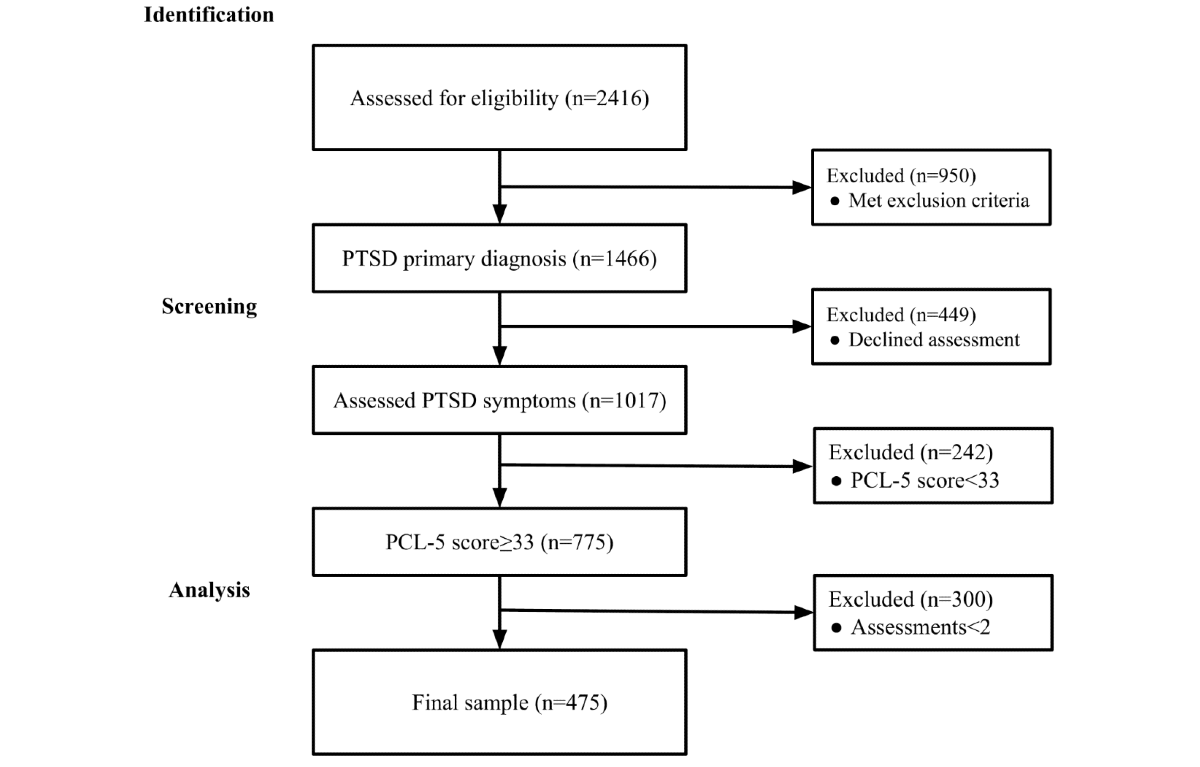
Flowchart of patient selection for the study. PCL-5: Posttraumatic Stress Disorder Checklist for the Diagnostic and Statistical Manual of Mental Disorders-5; PTSD: posttraumatic stress disorder.

Of 1466 individuals who had a confirmed PTSD diagnosis from licensed providers, 1017 did not meet the exclusion criteria and agreed to complete measures before beginning treatment; of these, 775 had PTSD symptoms above the clinical threshold. The final sample consisted of 475 participants who completed two or more surveys (Figure 1).

### Design and Procedure

Therapists in the study (n=173) were licensed in the same state as the patient and credentialed up to the National Committee for Quality Assurance standards. They were trained and experienced in PTSD (138/173, 79.7%), cognitive behavioral therapy (98/173, 56.9%), and third-wave behavioral (83/173, 47.7%) and psychodynamic (51/173, 29.4%) treatment modalities. They had at least three years of experience delivering mental health care post licensure. Modal experience of psychotherapy practice was 10+ years (69/173, 39.9%). Before providing treatment on the platform, therapists went through a 30-day orientation to the platform that included introduction to patients, completing informed consent, setting a frame for the treatment, appropriately pacing the treatment, handling crisis, making referrals, and other aspects related to telehealth competence.

Multimedia messages (similar in capability to commonly used *texting* apps) were used by the therapists to deliver interventions through a Health Insurance Portability and Accountability Act (HIPAA)–compliant interface for smartphones and computers. Messages consisted of two-way asynchronous communication, containing text, photo, audio, or video content. Therapists messaged patients a minimum of once (or more than once) per day, 5 days a week. Participants were able to send any number of multimedia messages at any time they wanted to their therapist, and the messages were stored for the clinicians to review. Therapist response times were scheduled and communicated to their patients at the start of treatment (eg, Monday to Friday from 9 AM to 11 AM and from 5 PM to 6 PM). All professional and ethical standards of the messaging treatment were observed just as in a face-to-face treatment, and higher levels of care referrals were provided when needed.

A period of 12 weeks of messaging was examined. Participants could discontinue treatment at any point as in conventional outpatient settings. Treatment engagement metrics included weekly average text, photo, audio, and video messaging communication for each week of the treatment. This information was automatically collected by the digital health platform. For each patient and therapist dyad, the average number of messages per week in treatment was assessed (text, audio, photo, and video) as well as the average number of weekly words used in text messages and the weekly average duration of audio and video content in minutes.

### Measures

All participants had a primary diagnosis of PTSD from their licensed provider. In addition, the 20-item PCL-5 [30,31] was used to identify the presence of PTSD symptoms from baseline and then every 3 weeks for 12 weeks. A PCL-5 score of 33 or higher is considered indicative of clinically significant PTSD [32], and only participants initially meeting the cutoff were included. Post baseline survey completion was voluntary and described to patients as an important aspect of their care that facilitates goal setting and tracking progress. In addition, the 12-item Working Alliance Inventory (WAI)–revised short patient version [33] determined participants’ treatment alliances after 3 weeks of treatment. WAI scores have been positively associated with good treatment outcomes [34].

### Statistical Analyses

#### Posttraumatic Stress Disorder Symptom Trajectories

Growth mixture modeling (GMM) was performed using Mplus 8 [35] to identify trajectories of PTSD symptom scores over treatment (baseline to week 12). GMM has been extensively used to model PTSD symptom course [36], as it allows the identification of heterogeneous subpopulations (or classes) characterized by different longitudinal symptom trajectories, such as treatment responders and nonresponders. The GMM trajectories were estimated using the Muthén-Roy model [37], a more stringent approach to survey nonresponse that considers missing data as nonignorable and associated with treatment measures. Specifically, binary indicators of missingness for each observation were included in the model and described by classes of missing data patterns, which correlated with the PTSD trajectories predicted by growth factors (intercept, slope, and quadratic parameters) of PCL-5 score changes over time. Patients were then assigned to one missing data pattern and a PCL-5 class, conjointly representing their treatment outcomes. To determine the best fitting model, nested GMMs with an increasing number of trajectories and different missing data approaches to dropout were compared [37]. The optimal trajectory solution [38] was determined based on the lower Bayesian Information Criterion and higher classification entropy [39]. Theoretical and explanatory properties and theoretical coherence were also considered when determining the best fitting solution [40].

#### Predictors of Posttraumatic Stress Disorder Reduction and Treatment Adherence

After determining the best fitting trajectory solution, categorical class assignments as well as demographic, clinical, and treatment variables were added into a logistic regression in R [41] to predict clinically meaningful PTSD symptom improvement, as indicated by the PCL-5 score reduction of 10 or more points [30] at the last available observation or at 12 weeks, whichever came first. A second logistic regression examined predictors of patient adherence, measured as remaining in treatment for at least eight weeks.

Analyzed predictors consisted of demographics (gender, age, and education level), first experience in therapy, total number of treatment weeks, working alliance (WAI score), therapist-reported PTSD treatment expertise, and years of experience. Weekly communication characteristics (types of messages, average words sent, and duration of audio/video messages) of therapist interventions and patient responses were also analyzed. Examined predictor variables had 12% missingness or less, with the exception of the WAI (32%). Multiple imputations by iterative random forests (500 trees, 10 iterations) for missing values were performed in R using package missForest v 1.4 [42]. Clinical and outcome variables were masked during imputations to prevent information leakage. Categorical variables were then transformed into binary (eg, age ≤ or >35 years).

## Results

### Sample Characteristics

The final patient sample consisted of 475 individuals with both PTSD diagnosis and PCL-5 scores of 33 or above. They were predominantly female, 26- to 35-year-old, and college educated. Table 1 reports the full characteristics of the participants.

**Table 1:** Demographic, clinical, and treatment characteristics for full sample (N=475).

Variable				Total number of participants		Value
**Age (years), n (%)**				475		
	18-25					125 (26.3)
	26-35					234 (49.3)
	36-49					103 (21.7)
	≥50					13 (2.7)
**Education, n (%)**				422		
	Bachelor’s degree or higher					291 (69.0)
	High school diploma					131 (31.0)
First time in therapy, n (%)						78 (17.8)
**Gender, n (%)**				475		
	Female					412 (86.7)
	Male					63 (13.3)
**Patient’s state, n (%)**				475		
	California					61 (14.2)
	New York					52 (12.1)
	Texas					28 (6.5)
	Florida					28 (6.5)
	Pennsylvania					20 (4.6)
	Other US state					347 (56.1)
**Posttraumatic stress disorder symptoms, mean (SD)**						
	PCL-5^a^, baseline			475		50.64 (10.44)
	PCL-5, week 3			475		43.53 (15.04)
	PCL-5, week 6			282		39.71 (15.80)
	PCL-5, week 9			168		37.65 (16.21)
	PCL-5, week 12			108		36.03 (16.80)
Treatment duration (weeks), mean (SD)				475		10.28 (2.82)
**Treatment focus, n (%)**				68		
	Traumatic memories					36 (53)
	Challenges with daily living					32 (47)
**Weekly engagement, patients, mean (SD)**				423		
	Number of messages					14.68 (18.04)
	**Text messages**					
		Count			13.78 (17.68)	
		Words			1039.86 (1191)	
	**Audio messages**					
		Count			0.40 (1.46)	
		Duration (min)			83.32 (330.93)	
	Photo messages, count					0.42 (1.05)
	**Video messages**					
		Count			0.05 (0.37)	
		Duration (min)			5.20 (41.86)	
**Weekly interventions by therapists, mean (SD)**				423		
	Number of messages					8.75 (8.88)
	**Text messages**					
		Count			8.18 (8.84)	
		Words			679.78 (535.12)	
	**Audio messages**					
		Count			0.33 (0.91)	
		Duration (min)			58.13 (180.48)	
	Photo messages, count					0.17 (0.49)
	**Video messages**					
		Count			0.07 (0.18)	
		Duration (min)			4.33 (17.33)	
Working alliance, mean (SD)				322		45.44 (10.47)

^a^PCL-5: Posttraumatic Stress Disorder Checklist for the Diagnostic and Statistical Manual of Mental Disorders-5.

### Treatment Characteristics

#### Messaging Engagement

Therapists predominantly delivered therapy via text on a weekly basis (mean 8.18, SD 8.84), but audio, photo, and video messages were also used. Similar communication preferences were observed in the patients’ weekly use of text (mean 13.78, SD 17.68), audio, photo, and video messages. A subset of therapists (68/173, 39.3%) described their treatment focus, with half reporting focusing on the memory of the trauma as the primary treatment goal (n=36) and the other on challenges with day-to-day living (n=32). Overall, patients reported good therapeutic alliance at 3 weeks of treatment (WAI, total scale: mean 45.44, SD 10.47; item average: mean 3.8, SD 0.9).

#### Dropout

The average treatment duration was 10.3 weeks (SD 2.7). A total of 58.7% (279/475) of patients completed the entire observed 12 weeks of treatment, with the remaining individuals discontinuing. Specifically, of the 475 patients, 11 (1.3% total) terminated treatment by week 3, 65 (13.7% total) terminated cumulatively by week 6, 127 (26.7%) terminated cumulatively by week 9, and an additional 69 discontinued before week 12, resulting in a total of 196 patients (41.3%) who discontinued treatment. Reasons for termination were reported in an exit survey of 108 individuals and included reaching personal treatment goals (n=52), considering the therapist not helpful or bad (n=23), money concerns (n=21), starting face-to-face therapy (n=9), lack of time (n=2), and technical difficulties with the platform (n=1). A therapeutic dose of PTSD treatment, consisting of 8 (or more) weeks of intervention, was achieved in 84.0% (399/475) of all patients.

#### Average Treatment Outcome

Mean PCL-5 scores averaged over the entire sample decreased from baseline (mean 50.64, SD 10.44) over the course of treatment: 3 weeks (mean 43.53, SD 15.04), 6 weeks (mean 39.71, SD 15.8), 9 weeks (mean 37.65, SD 16.21), and 12 weeks (mean 36.03, SD 16.8). There were 3.2 (SD 1.2) PCL-5 assessments available per patient. Scores falling below the established cutoff for probable PTSD (<33) were endorsed by 34.9% (166/475) of participants by their last observation; a more stringent threshold for remission based on the PCL-5 score of 19 and lower was reached by 14.3% (68/475) of the sample. The mean PCL-5 score reduction was 11 points (SD 14.47), with 48.6% (231/475) of patients reaching a clinically significant improvement of 10 or more points.

#### Posttraumatic Stress Disorder Symptom Trajectories

**Figure 2:**
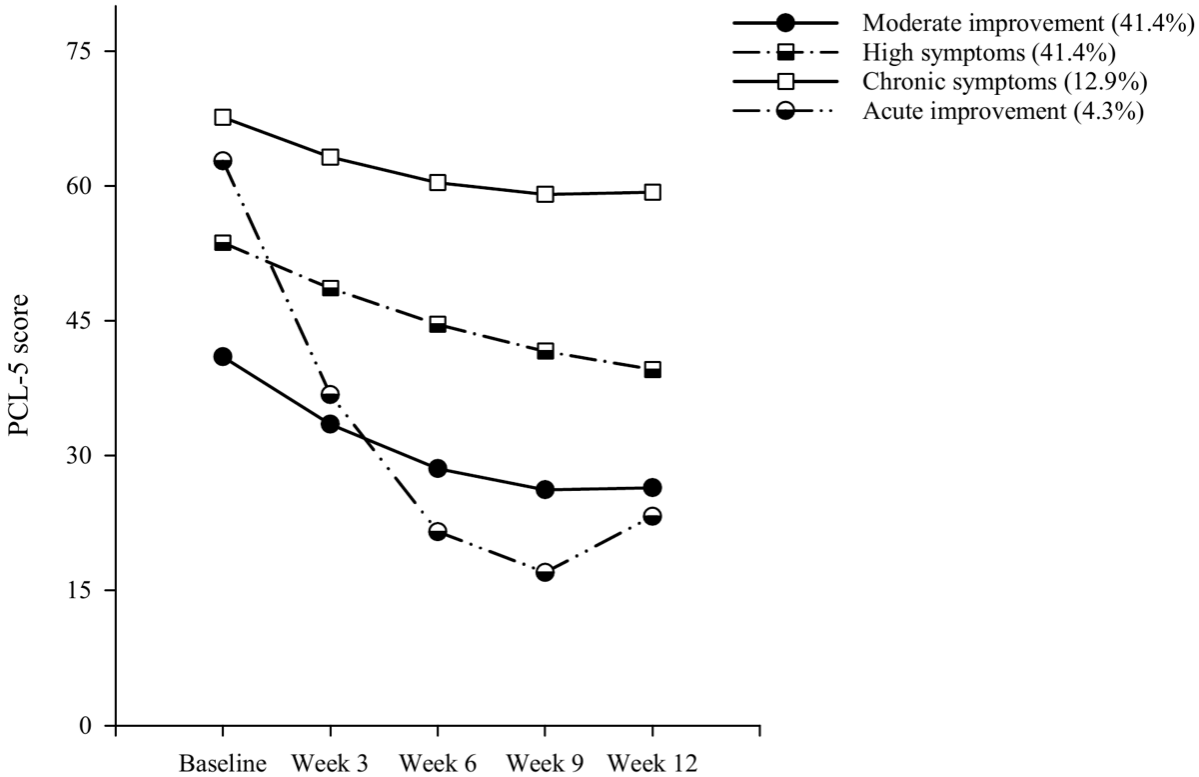
Estimated means for Muthén-Roy growth mixture modeling trajectories of PCL-5 symptom scores over 12 weeks of treatment (N=475). PCL-5: Posttraumatic Stress Disorder Checklist for the Diagnostic and Statistical Manual of Mental Disorders-5.

The best GMM fitting model of PCL-5 symptom trajectories over treatment is displayed in Figure 2. Table 2 reports fit indices for successfully estimated solutions of the GMM Muthén-Roy and other GMM models with progressively increasing numbers of classes.

GMM identified 4 subpopulations distinguished by their course of PCL-5 symptoms over treatment and their response patterns. The probability of distinct class membership was high, ranging from 0.78 to 0.92. The first trajectory was characterized by a steady reduction in symptom scores that eventually fell below the threshold established for probable PTSD (*moderate improvement*, 197/475, 41.4%). This class showed relatively lower levels of initial symptoms that decreased below the PTSD cutoff over the course of treatment. The second largest class (*high symptoms*, 197/475, 41.4%) described a population with PCL-5 scores that, although decreasing, remained above the clinical cutoff. The third identified class (*chronic symptoms*, 61/475, 12.9%) was a trajectory characterized by treatment nonresponse. The fourth trajectory (*acute improvement*, 20/475, 4.3%) was characterized by high initial PCL-5 scores, which decreased below the clinical cutoff through treatment. In terms of missing data patterns, the *moderate improvement* and *chronic symptoms* trajectories had the same latent profile, characterized by a higher risk of self-report measure noncompletion (44.0% of combined subpopulations completed only two measures) when compared with the *high symptoms* and *acute improvement* group (37.8%). There were no differences in dropout rates between the two missingness patterns (8+ weeks of treatment: 84.2% vs 83.8%). Consistent with their longitudinal course, the *moderate* and *acute improvement* trajectories belonged to the same PTSD latent class, characterized by symptom reduction, whereas *high* and *chronic symptoms* were part of the same elevated-symptom PTSD class. Specifically, of the 166 patients reaching scores below probable PTSD (PCL-5≤32) by their last observation, 130 were assigned to the symptom-reduction trajectories and 36 to the elevated-symptom class.

**Table 2:** Fit indices for Growth Mixture Models with increasing trajectory solutions.

Model and Number of classes		Bayesian Information Criterion	Entropy
**Muthén–Roy**			
	2 (2)	11,508.56	0.80
	1 (1)	11,556.90	0.70
**Pattern mixture**			
	N/A^a^	11,583.12	N/A
**Diggle-Kenward**			
	2	12,934.68	0.70
	1	13,092.78	N/A
**Missing at random GMM**			
	2	11,538.72	0.70
	1	11,724.37	N/A

^a^N/A: not applicable.

#### Predictors of Posttraumatic Stress Disorder Reduction and Treatment Adherence

Logistic regression analyses were performed to determine the role of demographic, categorical GMM memberships, treatment, and communication characteristics as predictors of significant PTSD reduction and treatment adherence.

The threshold for clinically meaningful PTSD symptom improvement (PCL-5 score reduction ≥10) was reached by 48.6% (231/475) of patients by their last observation, with 130 patients assigned to the *acute* and *moderate* *improvement* trajectories and 101 assigned to the *chronic* and *high symptom* trajectories. The likelihood ratio test of the full model against a constant-only model was significant (χ^2^_20,475_=64.1, *P<*.001; McFadden R²=0.10), and the predictor odds ratios (OR) are shown in Table 3. Results indicated that higher therapeutic alliance scores (OR 1.03, 95% CI 1.01-1.05; *P=*.02) and being at the first experience of psychotherapy (OR 2.03, 95% CI 1.18-3.54; *P=*.01) were significantly associated with PTSD symptom reduction. Consistent with the heterogeneous trajectories, the results also showed that participants assigned to the *moderate* and *acute* improvement trajectories were more likely to have significant symptom reduction (OR 5.19, 95% CI 2.83-10.0; *P<*.001) than those assigned to the elevated-symptom trajectories, while patients with less measure completion were also less likely to show improvements (OR 0.38, 95% CI 0.20-0.69; *P=*.002). In terms of average weekly engagement per week in treatment, patients sending more video messages per treatment week were more likely to show symptom improvements (OR 1.01, 95% CI 1.01-1.05; *P=*.03).

A second logistic regression examined predictors of staying in treatment until an adequate therapeutic dose (≥8 weeks), which was achieved by 84% (399/475) of patients. The analysis used all previously examined variables, with the exception of treatment duration. Results of the logistic regression (χ^2^_19,475_=118, *P<*.001; McFadden R²=0.28) indicated that patients with higher therapeutic alliances (OR 1.07, 95% CI 1.03-1.10; *P<*.001) and higher education level (OR 2.13, 95% CI 1.13-4.03; *P=*.02) were more likely to attain the treatment dose. Patients who were more engaged in treatment media who sent a higher number of messages per week of treatment were more likely to achieve treatment completion (OR 1.08, 95% CI 1.04-1.13; *P<*.001). Interestingly, therapists wrote more on average each week to patients who ended up discontinuing the therapy (OR 0.88, 95% CI 0.82-0.94; *P<*.001), possibly trying to keep them continuing the treatment. No other meaningful differences emerged in terms of covariates or quantitative intervention characteristics, and full estimates are reported in Table 3.

**Table 3:** Predictors of posttraumatic stress disorder symptoms clinically significant improvement and treatment adherence (N=475).

Variable		PTSD^a^ symptoms improvement		Treatment dose 8+ weeks	
		OR^b^ (95% CI)	*P* value	OR (95% CI)	*P* value
**Demographics**					
	Age>35 years	0.79 (0.49-1.26)	.32	0.91 (0.45-1.90)	.80
	Education: Bachelor’s degree or higher	0.99 (0.64-1.54)	.97	2.13 (1.13-4.03)	.02
	Gender - female	1.31 (0.73-2.36)	.37	0.69 (0.26-1.69)	.44
	First therapy experience	2.03 (1.18-3.54)	.01	0.96 (0.44-2.23)	.92
**Treatment** **characteristics**					
	Working alliance	1.03 (1.01-1.05)	.02	1.07 (1.03-1.10)	<.001
	Duration of treatment (weeks)	1.07 (0.98-1.17)	.13	N/A^c^	N/A
	Latent class: acute/moderate improvement	5.19 (2.83-10.0)	<.001	1.68 (0.72-3.92)	.23
	Latent class: survey noncompletion	0.38 (0.20-0.69)	.002	0.58 (0.25-1.34)	.20
	Therapist: years of experience (10+)	1.44 (0.92-2.27)	.11	0.68 (0.35-1.36)	.27
	Therapist: PTSD expertise	0.76 (0.51-1.14)	.19	0.84 (0.44-1.55)	.57
**Patient weekly engagement**					
	Number of messages	1.00 (0.98-1.01)	.64	1.08 (1.04-1.13)	<.001
	Text messages: number of words	1.00 (1.00-1.00)	.67	1.00 (1.00-1.00)	.07
	Audio messages: duration (min)	1.00 (1.00-1.00)	.52	1.00 (1.00-1.01)	.11
	Photo messages: count	0.83 (0.65-1.03)	.11	1.22 (0.82-2.14)	.40
	Video messages: duration (min)	1.02 (1.01-1.05)	.03	1.01 (0.99-1.04)	.47
**Therapist weekly engagement**					
	Number of messages	1.01 (0.97-1.05)	.55	0.88 (0.82-0.94)	<.001
	Text messages: number of words	1.00 (1.00-1.00)	.52	1.00 (1.00-1.00)	<.001
	Audio messages: duration (min)	1.00 (1.00-1.00)	.92	1.00 (0.99-1.00)	.15
	Photo messages: count	1.17 (0.74-1.99)	.51	1.43 (0.82-3.18)	.28
	Video messages: duration (min)	0.98 (0.95-1.00)	.05	0.99 (0.96-1.02)	.43

^a^PTSD: posttraumatic stress disorder.

^b^OR: odds ratio.

^c^N/A: not applicable.

## Discussion

This study analyzed two-way asynchronous messaging as a delivery modality for PTSD treatment. The research took place in a patient-centered naturalistic setting, where therapeutic dyads interacted daily through messaging. PTSD symptoms were assessed from baseline every 3 weeks over the course of 12 weeks of treatment. Messages were sent using a HIPAA-compliant online platform, which also automatically collected patient-therapist messaging engagement metrics. We first investigated whether PTSD treatment response patterns were influenced by the messaging delivery modality. We then divided patients into heterogeneous groups based on their symptom fluctuations to tease apart patterns of treatment response. We then assessed which individual, therapist, and messaging engagement differences could predict outcome differences.

The results indicated that 48.6% (231/475) of patients experienced clinically significant improvement of symptoms, with 34.9% (166/275) improving below the PTSD threshold. Similar remission rates are usually observed when delivering PTSD treatment through established modalities, including face-to-face [43,44] and live video [16,45]. In addition, we identified 4 heterogeneous trajectories of PTSD symptom changes. The majority of the sample showed a steady reduction in PTSD symptoms from a moderate baseline (*moderate improvement*, 198/475, 41.4%) and others more steeply from a higher baseline (*acute improvement,* 20/475, 4.3%). Nonetheless, one of the largest groups consisted of 198 individuals with symptoms that, while decreasing, remained above the clinical threshold (*high symptoms*, 198/475, 41.4%). A group of 12.9% (61/475) of patients with chronic high symptoms was also identified. The analytic approach allowed for a more accurate model of the heterogeneous PTSD course [46] and accounted for missing data in the course of treatment over time [37], identifying symptom improvements for nearly half of the sample in the examined time frame. These trajectories raise important possibilities for assisting the two nonresponder groups. For example, individuals in these groups could be referred to face-to-face therapy, switched to another type of therapy, moved to live video sessions, or offered a psychiatric referral or other available resources. Further analysis of participants with clinically meaningful PTSD symptom reductions indicated that the improvement trajectories were significantly more likely to reach PTSD remission. A higher chance of meaningful symptom reduction was also associated with being in therapy for the first time, a higher working alliance, higher survey response rate, and more use of immersive communication modalities (ie, video messages). Therapists’ expertise and the treatment focus were shown to be less important than other therapeutic factors (ie, working alliance). However, these findings may evolve, as therapeutic interventions were specifically identified for this medium. Of note, 84.0% (399/475) of the sample remained in treatment until reaching a therapeutic dose of 8+ weeks of therapy, a proportion higher than that observed in face-to-face PTSD treatment [43]. This finding is consistent with the 3 unique advantages of messaging therapy: (1) ease of access contributes to adherence and completion, (2) patients can write to therapists at any moment of the patient’s choosing, and (3) there is greater frequency of therapeutic intervention (5 days a week). Logistic regression analysis showed that treatment adherence was associated with a higher therapeutic alliance and more messages sent by patients each week. The role of working alliances in predicting treatment adherence and symptom improvement through messaging was consistent with its importance during in-person and live video therapy delivery [16,47,48]. Moreover, the therapists of individuals who discontinued treatment prematurely were more likely to try to engage their patients, resulting in higher average messages generated per week.

Overall, PTSD treatment administered using two-way messaging resulted in symptom-reduction rates similar to those reported for face-to-face and videoconferencing therapy [16,45] and higher spontaneous remission rates [49]. To our knowledge, this study, which observes the PTSD symptom course and treatment delivery in a large sample of patient-therapist dyads interacting through two-way messaging, is the first of its kind. Although these initial observational results are promising, the lack of a control group and posttreatment assessment in this study hinders comparison with nondigital treatment as usual. It may be useful to conduct a randomized controlled study of PTSD treatment delivery via asynchronous two-way messaging to assess its effectiveness compared with established forms of treatment interventions. Another limitation of the study is that our research was focused on the delivery of treatment rather than its content. Accordingly, the treatment metrics examined were related to the frequency (ie, number of messages) and quantity (ie, number of words and length of audio/video messages) of the patient-therapist interactions. Although we investigated therapist experience and PTSD treatment expertise as well as reported on treatment focus, our level of analysis was not specific to the content of treatment interventions. Future research utilizing treatment protocols or otherwise quantifying the nature of the therapeutic intervention will also be important. There are additional limitations to consider when interpreting these findings. Despite the large sample size in this study, 449 potential participants chose to not complete any assessments. In addition, 300 participants did not complete the minimum of two assessments required to be included in the study. As such, self-selection could have influenced the findings. Moreover, although encouraged by the platform and therapist, survey responses were determined by the patient, with possible reasons for nonresponse ranging from dissatisfaction (eg, unhappiness with their progress, lack of motivation, or discomfort with the modality) to treatment success (eg, met goals). Although the trajectory modeling used in this study accounted for missing data and dropout attrition, future research should assess these characteristics in more detail, in addition to the outcome findings discussed, to strengthen the generalizability of these results. Finally, although therapists received telehealth orientation, no PTSD intervention manuals for messaging were available at the time of the study. Future research should examine the feasibility and effectiveness of specific treatment approaches and protocols for PTSD when delivered by messaging, such as cognitive processing therapy.

Despite these limitations, this study highlights new opportunities for telemedicine in the treatment of PTSD. Patients diagnosed with PTSD often face avoidance, guilt, fear, and alienation in addition to common barriers (wait lists, insurance coverage, affordability, and scheduling around work or other obligations) that prevent them from seeking treatment. Delivery of therapy through two-way messaging opens up opportunities for increased access to treatment with less fear of stigmatization in an immediate and convenient manner.

## Abbreviations

GMM: growth mixture modeling
HIPAA: Health Insurance Portability and Accountability Act
MMS: multimedia message service
OR: odds ratio
PCL-5: Posttraumatic Stress Disorder Checklist for the Diagnostic and Statistical Manual of Mental Disorders-5.
PTSD: posttraumatic stress disorder
WAI: Working Alliance Inventory
